# Association between Sleep Duration, Social Jetlag, and the Metabolic Syndrome by Shift Works

**DOI:** 10.3390/ijerph21060668

**Published:** 2024-05-23

**Authors:** Minjung Kyung, Sungwon Park, Chang Gi Park, OiSaeng Hong

**Affiliations:** 1 School of Nursing, College of Nursing, The Catholic University of Korea, 222 Banpo-daero, Seocho-gu, Seoul 06591, Republic of Korea; 2 School of Nursing, University of Michigan, 400 North Ingalls Street, Ann Arbor, MI 48109, USA; rebecca.sungwon.park@gmail.com; 3 College of Nursing, University of Illinois at Chicago, 845 S. Damen Ave., MC 802, Chicago, IL 60612, USA; parkcg@uic.edu; 4 School of Nursing, University of California, San Francisco, 2 Koret Way, San Francisco, CA 94143, USA; oisaeng.hong@ucsf.edu

**Keywords:** lifestyle factors, metabolic syndrome, shift work, sleep duration, social jetlag

## Abstract

Lifestyle factors, including sleep characteristics, have been implicated in the development of metabolic syndrome, particularly among shift workers. This study aimed to explore the relationship between shift work, sleep duration, social jetlag, and the risk of metabolic syndrome among U.S. workers and the moderating effect of sleep duration and social jetlag on this relationship. Data from the National Health and Nutrition Examination Survey (NHANES) in 2017–2020 March were analyzed. Poisson regression models were employed to examine associations. Among 4136 U.S. workers, 53.3% had metabolic syndrome, with a higher proportion of shift workers (63.8% vs. 56.7%, *p* = 0.001) and those sleeping less than 6 h or more than 9 h per week (22.3% vs. 19.1%, *p* = 0.044) in the affected group. Shift workers were initially found to have an increased risk of metabolic syndrome (Coef. = 0.03, 95% CI: 0.02, 0.16); however, this association was mitigated when accounting for the interaction with social jetlag. Specifically, 1 to <2 h of social jetlag interacted significantly, increasing metabolic risk (Coef. = 0.15, 95% CI: 0.09, 0.22), whereas 1 to <2 h alone showed a protective effect (Coef. = −0.11, 95% CI: −0.17, −0.06). These findings suggest that optimizing sleep schedules and addressing social jetlag may be crucial in mitigating metabolic syndrome risks among shift workers.

## 1. Introduction

Metabolic syndrome represents a significant global health concern [1]. Over the decades, its prevalence has exhibited a concerning upward trajectory, surging from 25.3% in 1988 to 38.3% in 2018 in the United States [2,3]. This concerning trend is reflective of the epidemiological transition accompanying rapid economic growth, with global prevalence estimates varying from 12.5% to 31.4% depending on the operational definition employed [1]. Initially confined to the Western world, metabolic syndrome has evolved into a global issue that transcends geographical boundaries [4]. Its prevalence is often higher in the urban populations of certain developing nations compared to their Western counterparts [4]. Also known as insulin resistance syndrome, metabolic syndrome is characterized by a cluster of cardiometabolic risk factors, including abdominal obesity, high blood pressure, increased fasting glucose levels, elevated triglycerides, and low high-density lipoprotein cholesterol (HDL) levels [2]. Many studies have established that metabolic syndrome significantly amplifies the risk of developing type 2 diabetes and cardiovascular diseases [5]. A prospective cohort study further emphasized the severity of metabolic syndrome, revealing a notable increase in the risk of both cardiovascular and all-cause mortality as the number of metabolic syndrome risk factors rose [6].

Several lifestyle factors, including sleep patterns, dietary choices, levels of physical activity, sedentary behavior, alcohol consumption, and smoking habits, have been identified as risk factors contributing to the development of metabolic syndrome [7,8]. Among these, sleep characteristics such as sleep duration and social jetlag exert a profound and extensive influence on circadian rhythms and metabolism [9,10]. Inadequate sleep can lead to alterations in intermediate biological mechanisms, including inflammation, insulin sensitivity, hormone regulation, and the autonomic nervous system [11]. Both short and long sleep durations have been independently linked to unhealthy behaviors, such as poor dietary choices, physical inactivity, and weight gain [12]. Furthermore, social jetlag, which refers to the misalignment between an individual’s biological and social rhythms, has emerged as a potential risk factor for metabolic syndrome [11,13,14]. This circadian misalignment may have an adverse effect on the gut microbiome, glucose regulation, and inflammation, both directly and indirectly contributing to detrimental changes in the cardiovascular system [15]. Thus, the intricate interplay between sleep patterns, circadian rhythms, and metabolic health underscores the importance of considering sleep quality and timing as integral components of lifestyle interventions aimed at preventing or managing metabolic syndrome.

Sleep may play a role in the emergence of metabolic syndrome, particularly among workers engaged in shift work [16]. Shift work, characterized by non-traditional work hours outside the typical 9am to 5pm framework, includes schedules spanning early morning, evening, nights, and rotating shifts. This form of employment, now embraced by approximately a quarter of the global workforce, has steadily risen in prevalence in response to evolving social demands [17,18,19,20]. The consequences of shift work extend beyond mere disruption of circadian rhythms, resulting in a wide array of adverse health outcomes. These include an increased risk of developing metabolic syndrome, heightened susceptibility to diseases such as cancer and mental health disorders, and even elevated vulnerability to infections like COVID-19 [21,22]. A meta-analysis examining the association between shift work and the risk for metabolic syndrome among healthcare workers revealed that shift workers had more than a twofold increase in the likelihood of developing metabolic syndrome [20]. However, shift workers often face challenges in achieving quality sleep [23]. While shift workers have similar overall sleep duration to non-shift workers, they tend to have more sleep periods that are either shorter and/or longer compared to non-shift workers [23]. Social jetlag is also common in shift workers due to the need to synchronize their circadian rhythm with the light-dark cycle, making it challenging for them to adjust to imposed sleep and activity schedules [24]. It was believed that the increased risk of metabolic syndrome among shift workers could be primarily attributed to an unhealthy lifestyle and lower socioeconomic status. However, a growing body of research now suggests that social jetlag serves as an independent risk factor for the development of metabolic syndrome [25].

The significance of understanding the influence of sleep duration and social jetlag on the development of metabolic syndrome among shift workers cannot be overstated, particularly considering that sleep hygiene can be modifiable. This understanding implies that lifestyle modifications can potentially mitigate the risk factors associated with metabolic syndrome in shift workers. While many studies have delved into the correlation between sleep characteristics, shift work, and metabolic syndrome [16], there remains a notable gap in the existing literature. Specifically, there is a scarcity of research that simultaneously examines the relationship between sleep duration and social jetlag, as well as their potential moderating effect on the relationship between shift work and the risk of metabolic syndrome. Addressing this gap is crucial for gaining a comprehensive understanding of the complex interplay between sleep patterns, occupational demands, and metabolic syndrome outcomes among shift workers.

Therefore, this study aimed (1) to examine the association between shift work, sleep duration, and social jetlag and the risk of metabolic syndrome among U.S. adult workers and (2) to investigate whether sleep duration and social jetlag moderate this relationship.

## 2. Participants and Methods

### 2.1. Study Design, Settings, and Participants

This study employed a cross-sectional design, utilizing data from the National Health and Nutrition Examination Survey (NHANES) conducted between 2017 and March 2020, pre-pandemic. NHNAES is a comprehensive survey conducted by the National Center for Health Statistics at the Centers for Disease Control and Prevention in 2-year cycles to assess the health and nutritional status of residents in the United States. It involves in-home interviews and physical examinations of a nationally representative, multistage stratified sample of non-institutionalized U.S. civilians. The dataset comprises sociodemographic, dietary, and health-related information, including medical histories, dental assessments, physiological measurements, and comprehensive laboratory test results [26]. Prior to participation, informed consent was obtained from all survey participants, and all survey protocols received approval from the National Center for Health Statistics Ethics Review Board. IRB approval was waived as the data used is publicly available.

The initial cohort for analysis comprised 5590 participants aged 18 years or older, who completed medical examinations related to metabolic syndrome. Following a stringent selection process, 1454 participants were subsequently excluded from the analysis due to their non-working status at the time of survey administration based on the following criteria: individuals looking for a job (n = 174), those not engaged in any job or business (n = 1279), and those with uncertain work status (n = 1). Consequently, the final sample for this study was 4136 workers, with 2554 shift workers and 1582 non-shift workers.

### 2.2. Study Variables and Measures

The independent variable in this study was shift work status. Participants were asked to report their overall work schedule for the past three months. These responses were dichotomized into two groups: non-shift workers (adhering to traditional 9am to 5pm daytime schedules) and shift workers (encompassing early mornings, evenings, or nights) [27].

The moderating variables included usual weekday sleep duration and social jetlag. Participants were asked to indicate their average hours of sleep from Monday to Friday or workdays as their weekday sleep duration and their average hours of sleep from Saturday to Sunday or non-workdays as their weekend sleep duration. Sleep duration was categorized into three groups: (1) short sleep duration (less than 6 h), (2) normal sleep duration (6 h to less than 9 h), and (3) long sleep duration (9 h or more) [28]. In addition, another moderating variable, social jetlag, was calculated, and the absolute difference between mid-point sleep duration on weekdays and weekends was calculated. Social jetlag was also categorized into three groups: (1) normal social jetlag (less than 1 h), (2) moderate social jetlag (1 h to less than 2 h), and (3) severe social jetlag (2 h or more). [29].

The outcome variable was the number of metabolic syndrome components defined by the National Cholesterol Education Program Adult Treatment Panel Ⅲ criteria [30]. The National Cholesterol Education Program Adult Treatment Panel Ⅲ criteria encompassed various parameters indicative of metabolic dysfunction, including: (1) waist circumference over 40 inches for men or 35 inches for women, (2) blood pressure over 130/85 mmHg, (3) fasting triglyceride over 150 mg/dL, (4) fasting HDL cholesterol less than 40 mg/dL for men or 50 mg/dL for women, and (5) fasting glucose over 100 mg/dL [30]. The number of metabolic syndrome components ranged from 0 to 5, with a diagnosis of metabolic syndrome requiring the presence of at least three of these established criteria.

Covariates included in the analysis comprised demographic, socioeconomic, lifestyle, and health-related factors known to influence metabolic syndrome. These covariates were age, sex, race/ethnicity (non-Hispanic White, non-Hispanic Black, Hispanic or Latino, non-Hispanic Asian, and others, including multi-racial), education level (less than high school, high school diploma/general educational development test (GED), and some college or above), marital status (married/living with partner, widowed/divorced/separated, and never married), income-to-poverty ratio, alcohol consumption, smoking, physical activity, body mass index (BMI), and healthy diet. The selection of these covariates was based on previous research, particularly drawing upon the findings of a recent systematic review and meta-analysis [12]. Alcohol consumption patterns were dichotomized into two groups: non-to-moderate (drinking alcohol less than 5 days per week) and heavy drinkers (drinking alcohol 5 days or more per week), following the definition provided by the United States Substance Abuse and Mental Health Services Administration [27]. Smoking status was assessed using two questions regarding participants’ lifetime smoking history and current smoking status: whether participants have ever smoked at least 100 cigarettes and whether they currently smoke cigarettes [31]. Participants were categorized into one of three groups: non-smokers (those who have not smoked 100 cigarettes in their lifetime and do not currently smoke), former smokers (those who have smoked 100 cigarettes in their lifetime but do not currently smoke), and current smokers (those who have smoked 100 cigarettes in their lifetime and currently smoke) [31]. Physical activity was calculated by multiplying the duration and frequency of both moderate and vigorous activities. To achieve this, one minute of vigorous activity was considered equivalent to two minutes of moderate activity. Subsequently, the total physical activity was dichotomized into two groups based on a cutoff of 150 min per week: inadequate physical activity (less than 150 min per week) and adequate physical activity (150 min per week or more) [31]. The assessment of a healthy diet relied on a single question asking participants to rate the overall healthiness of their diet on a 5-point Likert scale. Higher scores indicated poorer dietary habits (1 = excellent, 2 = very good, 3 = good, 4 = fair, 5 = poor).

### 2.3. Data Analysis

The data were analyzed using STATA version 16.0 (Stata Corp., College Station, TX, USA). Descriptive statistics, including unweighted frequencies, weighted proportions, means, and standard deviation (SD), were used to summarize the sample characteristics. Differences in the sample characteristics were assessed by shift work using Chi-square tests and two-tailed t-tests with a 95% confidence interval. Chi-square tests were also employed to compare shift work status and sleep characteristics by metabolic syndrome. Following assessment for data overdispersion, Poisson regression with a robust variance estimation was carried out to examine the sleep characteristics, shift work, and the risk of metabolic syndrome. The potential moderating effect of sleep characteristics on this relationship was also investigated. After evaluating for multicollinearity, variables with a *p* of less than 0.05 from the bivariate analysis were included in the multivariable analyses. Model 1 was adjusted for variables such as age, sex, race, education, marital status, ratio of family income to poverty, physical activity, BMI, smoking, and healthy diet. Model 2 introduced an additional adjustment for the interaction between shift work and sleep duration, while Model 3 further included the interaction between shift work and social jetlag from Model 1. The coefficient, robust standard error, and 95% confidence intervals (CI) were calculated as associate measures.

## 3. Results

### 3.1. Characteristics of the Study Participants

The characteristics of the study participants are summarized in Table 1. Out of 4136 participants, 2544 (61.5%) were shift workers, and 2153 (50.7%) were female. The weighted mean age of the participants was 46.6 years. The majority of participants were non-Hispanic White (67.7%), had some college education or above (71.2%), and were married or living with a partner (63.5%). The mean ratio of income to poverty was 3.1 out of 5, with non-shift workers at 3.5 and shift workers at 2.9. Shift workers exhibited a lower proportion of females (50.0% vs. 51.7%, *p* = 0.002), non-Hispanic Whites (66.8% vs. 69.2%, *p* < 0.001), workers with some college or above education level (68.4% vs. 75.5%, *p* < 0.001), and those married or living with a partner (62.2% vs. 65.5%, *p* = 0.004) compared to non-shift workers. Shift workers included a higher proportion of current or former smokers (45.3% vs. 33.5%, *p* < 0.001) and those with inadequate physical activity less than 150 min per week (76.2% vs. 72.4%, *p* < 0.001), compared to non-shift workers. Shift workers also had a slightly higher BMI (29.8 vs. 29.2, *p* = 0.006) and a lower proportion reporting an excellent or very good diet (32.5% vs. 35.6%, *p* < 0.001). The average number of metabolic risk factors was 2.6 for all workers, but it was significantly higher among shift workers compared to non-shift workers (2.6 vs. 2.5, *p* < 0.001). Furthermore, a larger proportion of shift workers exhibited three or more risk factors for metabolic syndrome compared to non-shift workers (55.3% vs. 48.0%, *p* < 0.001).

### 3.2. Shift Work and Sleep Characteristics of Metabolic Syndrome

Table 2 presents shift work and sleep characteristics by metabolic syndrome for the participants. Based on the National Cholesterol Education Program Adult Treatment Panel Ⅲ criteria, risk factors for metabolic syndrome were categorized into two groups using a cutoff of three risk factors. About 53.5% of the participants had metabolic syndrome. Compared to the non-metabolic syndrome group, the metabolic syndrome group had a higher proportion of shift workers (63.8% vs. 56.7%, *p* = 0.001) and workers who slept less than 6 h or 9 h or more during the week (22.3% vs. 19.1%, *p* = 0.044). While workers with metabolic syndrome also showed a greater prevalence of two hours or more of social jetlag compared to those without metabolic syndrome, this difference did not reach statistical significance (28.1% vs. 23.5%, *p* = 0.072).

### 3.3. Association between Shift Work, Sleep Characteristics, and the Risk of Metabolic Syndrome

Table 3 provides an association between shift work, sleep characteristics, and the risk of metabolic syndrome. In a multiple Poisson regression (Model 1) with robust variance, shift workers showed a higher risk of metabolic syndrome compared to non-shift workers (coefficient = 0.03, 95% CI: 0.02, 0.16), after adjusting for age, sex, race/ethnicity, education, marital status, income to poverty ratio, physical activity, BMI, smoking habit, healthy diet, and sleep characteristics. There was no statistically significant association observed between sleep characteristics and the risk of metabolic syndrome. Upon further adjustment for the interaction between shift work and sleep duration in Model 2, the significance of shift work persisted (coefficient = 0.05, 95% CI: 0.02, 0.08), while the association between sleep characteristics and the risk of metabolic syndrome remained statistically non-significant. In Model 3, additional adjustments were made for the interaction between shift work and social jetlag building upon Model 1. No direct association was found between shift work and the risk of developing metabolic syndrome. However, a positive association was observed when shift work interacted with having 1 to less than 2 h of social jetlag, which increased the risk of metabolic syndrome (coefficient = 0.15, 95% CI: 0.09, 0.22). In contrast, experiencing 1 to less than 2 h of social jetlag alone was associated with a decreased risk of metabolic syndrome (coefficient = −0.11, 95% CI: −0.17, −0.06).

## 4. Discussion

Using large amounts of data from the population-representative cohort of U.S. workers, this study examined the association between shift work, sleep duration, social jetlag, and the risk of metabolic syndrome and investigated whether sleep duration and social jetlag have a moderating effect on this relationship. The study found that 53.3% had metabolic syndrome. This prevalence exceeds that observed in the U.S. adult population aged 20 years or older (41.8%) and in the South Asian adult population aged 18 years or older (32.5%), as defined by the National Cholesterol Education Program Adult Treatment Panel Ⅲ criteria [3,32]. Within the United States, the prevalence of metabolic syndrome is reported to be highest among Hispanics and lowest among African Americans [33]. This variance can be attributed to both an increasing trend in metabolic syndrome and the intricate interplay of factors related to countries and race/ethnicity. Various governmental, institutional, and sociocultural factors vary across countries, influencing a spectrum of upstream determinants [34]. These factors encompass the availability and accessibility of foods, healthcare policies, educational opportunities, employment scenarios, and the physical environment [34]. Additionally, individual factors such as biology, genetics, and sociocultural aspects play significant roles, contributing to the diverse prevalence of metabolic syndrome between countries [34]. These diverse aspects collectively contribute to what is often termed ethnic or racial differences, as certain characteristics tend to cluster within specific and identifiable populations [34]. Even within the same country and local environment, the prevalence of metabolic syndrome varies among predefined racial or ethnic groups [34]. The findings of this study also shed light on a significant association between shift work and the heightened risk of metabolic syndrome, with an intriguing twist—social jetlag appears to play a moderating role in this relationship. No such association was observed between sleep duration and the risk of metabolic syndrome.

This study found an association between shift work and the risk of metabolic syndrome, which corroborates the findings of earlier studies [16,35,36,37]. Importantly, this significant association remained intact even after adjusting for sleep characteristics. In a meta-analysis, shift workers had 1.11 times higher odds of developing metabolic syndrome compared to non-shift workers in an adjusted model including sleep duration and quality [16]. A retrospective case–control study also reinforced this significant association by controlling for sleep duration as a potential confounding factor. What makes this study intriguing is that the significant impact of shift work on the risk of metabolic syndrome disappeared when further adjustment of the interaction between shift work and social jetlag was made. This may mean that the influence of shift work on the risk of metabolic syndrome is contingent upon an individual’s level of social jetlag. These findings highlight the importance of personalized health management, such as sleep hygiene and workplace policies tailored to the unique circumstances of each shift worker.

Whereas our study did not find a significant association between social jetlag and the risk of metabolic syndrome, 1–2 h of social jetlag was linked to a reduced risk of metabolic syndrome among non-shift workers after additional adjustment for the interaction between shift work and social jetlag. Earlier studies reported that higher social jetlag was associated with an increased likelihood of higher waist circumference, a component of metabolic syndrome [38,39]. One plausible reason for this result may be the comprehensive adjustment for various factors in our analysis, including shift work status, sleep duration, and the interaction between social jetlag and shift work as a potential confounding factor. Metabolic syndrome is a complex disorder influenced by a multitude of factors, necessitating a careful consideration of interactions between variables and the presence of confounding factors. This finding suggests that a slight misalignment between an individual’s work schedule and their natural circadian rhythms might have some protective effects against the development of metabolic syndrome. This result underscores the need for further research to unravel the underlying mechanisms and provide more tailored health recommendations based on work schedules.

Our study did not find any significant association between sleep duration and the risk of metabolic syndrome. This is consistent with prior research that reported that the effect of sleep duration on metabolic syndrome risk disappeared after accounting for potential confounding factors, including lifestyle factors, sleep quality, and shift work status [35,36,37]. This finding suggests that various confounding variables, known to influence metabolic syndrome, might have been masking the relationship between sleep duration and the risk of metabolic syndrome. In essence, the impact of sleep duration on metabolic health may be more subtle and intricate, with other factors playing a more dominant role. Consequently, this finding emphasizes the need for a holistic approach when studying metabolic syndrome risk among shift workers. Rather than isolating a single factor, such as sleep duration, we should consider a wide range of lifestyle and work-related factors. A comprehensive understanding of metabolic syndrome risk in this population requires an examination of the interplay among these factors.

Our research benefited from a nationally representative sample from the U.S. working population, which strengthens the robustness and generalizability of our findings. However, this study has several inherent limitations. First, this study focused on individuals who had been involved in shift work within the past three months. This selection criterion introduces the potential for the ‘healthy worker survivor effect’, whereby those excluded from our analysis may have had a higher risk of metabolic syndrome. Consequently, this may lead to an underestimation of the effects attributed to both shift work and sleep characteristics. Second, as a limitation of our secondary analysis of existing data, our investigation did not encompass an evaluation of critical dimensions of sleep, including sleep quality, individual chronotype, and the duration of involvement in shift work—factors previously associated with metabolic syndrome. The absence of these variables in this analysis may result in an oversimplified or biased interpretation of our findings. Third, due to the self-reported measures of sleep characteristics, shift work, and social jetlag, we cannot rule out recall or reporting bias. Fourth, the cross-sectional design employed in this study limits the ability to demonstrate causality between shift work, sleep characteristics, and the risk of metabolic syndrome.

## 5. Conclusions

This study of a population-based sample of workers revealed a potential significant association between shift work and metabolic syndrome risk. It also provided evidence that social jetlag, a modifiable factor, played a moderating role in this relationship. These findings highlight the importance of personalized health management, particularly for shift workers, which could involve optimizing sleep schedules while taking into account the extent of social jetlag. Such measures can play a crucial role in reducing the risk of metabolic syndrome. By adopting a holistic approach that integrates considerations of both sleep duration and social jetlag, more effective strategies for mitigating the risk of metabolic syndrome among shift workers can be devised. Implementing such measures not only promotes their well-being but also contributes to overall health improvement. Further research efforts should focus on elucidating the underlying mechanisms through which social jetlag influences metabolic syndrome among non-shift workers, while also employing improved measurement techniques to account for potential confounding factors such as sleep quality. This will further enhance our understanding of the intricate interplay between sleep patterns, social rhythms, and metabolic syndrome outcomes, facilitating the development of targeted interventions aimed at improving health outcomes for all workers.

## Figures and Tables

**Table 1:** Sample characteristics of U.S. workers: Analysis of NHANES 2017–March 2020.

Characteristics	U.S. Workers (N = 4136)		Non-Shift Workers (n = 1582)		Shift Workers ^a^ (n = 2554)		*p* *
	N	Weighted % (95% CI)	N	Weighted % (95% CI)	N	Weighted % (95% CI)	
Age, years (Mean, SD)	46.6	15.2	46.6	14.0	46.7	15.8	0.898
Sex							**0.002**
Male	1983	49.3 (44.4–54.2)	709	48.3 (38.1–58.6)	1274	50.0 (44.2–55.8)	
Female	2153	50.7 (45.7–55.6)	873	51.7 (41.4–61.9)	1280	50.0 (44.2–55.8)	
Race/ethnicity							**<0.001**
Non-Hispanic White	1488	67.7 (62.3–72.7)	574	69.2 (62.4–75.3)	914	66.8 (60.3–72.7)	
Non-Hispanic Black	804	7.4 (5.6–9.8)	270	6.7 (4.9–9.1)	534	7.9 (5.8–10.7)	
Hispanic or Latino	1028	15.5 (11.9–19.9)	360	13.9 (10.5–18.2)	668	16.5 (12–22.2)	
Non-Hispanic Asian	608	5.9 (3.9–8.6)	315	7.7 (4.9–12)	293	4.7 (3.1–6.8)	
Other including multi-racial	208	3.5 (2.5–4.8)	63	2.5 (1.4–4.1)	145	4.2 (2.8–6.1)	
Education level							**<0.001**
Less than high school	486	7.1 (5.5–9)	117	4.3 (2.8–6.5)	369	8.8 (6.6–11.9)	
High school diploma/GED	792	21.7 (18–26)	289	20.2 (14.8–27)	503	22.7 (18.7–27.3)	
Some college or above	2750	71.2 (67.3–74.8)	1149	75.5 (68.9–81)	1601	68.4 (63.9–72.6)	
Missing	108						
Marital status							**0.004**
Married/Living with partner	2570	63.5 (55.8–70.5)	1038	65.5 (57.6–72.7)	1532	62.2 (51.7–71.6)	
Widowed/Divorced/Separated	748	20.5 (15.7–26.4)	276	20.5 (14.2–28.7)	472	20.5 (14.1–28.9)	
Never married	710	16.0 (12.8–19.7)	241	14.0 (10.1–19)	469	17.3 (13.3–22.3)	
Missing	108						
Ratio of income to poverty ^b^ (Mean, SD)	3.1	1.6	3.5	1.6	2.9	1.6	**<0.001**
Alcohol use of (≥5 drinks per month							0.058
None to moderate (<5 days)	2843	92.6 (88.5–95.3)	1131	92.6 (84–96.8)	1712	92.5 (88.1–95.4)	
Heavy (≥5 days)	222	7.4 (4.7–11.5)	74	7.4 (3.2–16)	148	7.4 (4.6–11.9)	
Missing	1071						
Smoking status ^c^							**<0.001**
Current smoker	570	13.1 (11.1–15.4)	171	11.6 (7.7–17.2)	399	14.0 (11.2–17.5)	
Former smoker	1002	27.6 (24.8–30.6)	344	21.9 (17–27.8)	658	31.3 (28–34.8)	
Non-smoker	2559	59.3 (56–62.6)	1067	66.4 (59.8–72.5)	1492	54.7 (49.7–59.6)	
Missing	5						
Physical activity ^d^							**<0.001**
Inadequate (<150 min/week)	1225	25.3 (22.1–28.9)	528	27.6 (22.5–33.4)	697	23.8 (19.7–28.5)	
Adequate (≥150 min/week)	2911	74.7 (71.1–77.9)	1054	72.4 (66.6–77.5)	1857	76.2 (71.5–80.3)	
BMI (Mean, SD)	29.6	7.4	29.2	7.0	29.8	7.6	**0.006**
Self-reported healthy diet							**<0.001**
Excellent	314	8.7 (6.2–12.1)	122	8.3 (4.8–14.1)	192	9.0 (5.9–13.5)	
Very good	958	25.0 (19.7–31.1)	453	27.3 (20–36)	505	23.5 (16.9–31.7)	
Good	1509	38.1 (33.1–43.4)	575	36.8 (30.5–4.4)	934	38.9 (31.8–46.6)	
Fair	1035	23.7 (20.2–27.6)	333	23.5 (17.9–30.1)	702	23.8 (20.2–27.8)	
Poor	212	28.2 (24.7–32)	72	4.1 (2.8–6)	140	4.8 (3.7–6.2)	
Missing	108						
Number of metabolic risk factors ^e^ (Mean, SD)	2.6	1.2	2.5	1.2	2.6	1.2	**<0.001**
0	150	3.7 (2.4–5.7)	53	4.8 (2.4–9.6)	97	3.0 (2.1–4.3)	
1	698	18.7 (15.9–21.8)	304	22.1 (18.3–26.4)	394	16.4 (12.3–21.4)	
2	1082	25.2 (22.4–28.2)	432	25.1 (20.1–30.3)	650	25.3 (21.6–29.4)	
3	1193	28.4 (24.1–33)	461	27.2 (21–34.3)	732	29.2 (24.8–28.3)	
4	796	19.6 (16.2–23.7)	247	16.0 (12.3–20.5)	549	22.1 (16.9–28.3)	
5	217	4.4 (3.2–5.9)	85	4.8 (3.5–6.7)	132	4.0 (2.4–6.7)	

SD, standard deviation; * Numbers in bold indicate significant at *p* < 0.05; ^a^. Shift work includes evening or night, early mornings, and variable (early morning, days, and nights); ^b^. A higher value indicates higher socioeconomic status (range 0–5); ^c^. Smoking status was grouped as non-smokers who have not smoked 100 cigarettes in their lifetime and do not smoke now, former smokers who have smoked 100 cigarettes in their lifetime but do not smoke now, and current smokers who have smoked 100 cigarettes in their lifetime and smoke now; ^d^. Physical activity is calculated by multiplying the duration and frequency of moderate and vigorous activity, where one minute of vigorous activity are the equivalent to two minutes of moderate activity; ^e^. Based on the National Cholesterol Education Program Adult Treatment Panel III criteria (1) waist circumference over 40 inches for men or 35 inches for women, (2) blood pressure over 130/85 mmHg, (3) fasting triglyceride over 150 mg/dL, (4) fasting HDL cholesterol less than 40 mg/dL for men or 50 mg/dL for women, and (5) fasting glucose over 100 mg/dL.

**Table 2:** Shift work and sleep characteristics in the sample by metabolic syndrome.

Characteristics	U.S. Workers (N = 4136)		Without Metabolic Syndrome (n = 1930)		With Metabolic Syndrome (n = 2206)		*p* *
	N	Weighted % (95% CI)	N	Weighted % (95% CI)	N	Weighted % (95% CI)	
Shift workers	2554	60.4 (55.4–65)	1141	56.7 (49–64.1)	1413	63.8 (59.6–67.7)	**0.001**
Weekday sleep duration							**0.044**
<6 h	447	8.7 (6.4–11.7)	187	7.3 (4.9–10.9)	260	10.0 (6.6–14.7)	
6 to <9 h	3114	79.2 (75.7–82.4)	1485	80.9 (75.4–85.3)	1629	77.7 (72.4–82.2)	
≥9 h	575	12.1 (10.3–14.1)	258	11.8 (8.8–15.7)	317	12.3 (9.4–16)	
Weekend sleep duration							0.878
<6 h	216	4.5 (3–6.6)	104	4.9 (2.6–9.1)	112	4.1 (2.6–6.5)	
6 to <9 h	2302	60.7 (56.7–64.6)	1076	62.1 (55.1–68.7)	1226	59.4 (54–64.6)	
≥9 h	1618	34.8 (30.9–38.9)	750	33.0 (27.2–39.3)	868	36.5 (30.8–42.6)	
Social jetlag ^a^, hours							0.072
<1 h	1681	44.6 (40.2–49.1)	795	46.9 (41.5–52.4)	886	42.5 (37–48.2)	
1 to <2 h	1237	29.5 (24.9–34.5)	599	29.6 (24.2–33.6)	638	29.4 (24.5–34.9)	
≥2 h	1218	25.9 (21.3–31.1)	536	23.5 (18.9–29)	682	28.1 (21.7–35.5)	

Note: metabolic syndrome was categorized into two groups using a cutoff of three risk factors based on the National Cholesterol Education Program Adult Treatment Panel III criteria; * Numbers in bold indicate significant at *p* < 0.05; ^a^. The absolute difference between midpoint sleep duration on weekdays and weekends.

**Table 3:** Associations of shift work, sleep characteristics, and the risk of metabolic syndrome.

	Model 1			Model 2			Model 3		
	Coef	Robust SE	95% CI	Coef	Robust SE	95% CI	Coef	Robust SE	95% CI
Shift work	**0.03**	**0.01**	**0.02, 0.16**	**0.05**	**0.01**	**0.02, 0.08**	−0.02	0.02	−0.06, 0.02
Weekday sleep duration									
<6 h	0.03	0.02	−0.02, 0.07	0.07	0.04	−0.01, 0.16	0.03	0.02	−0.01, 0.08
6 to <9 h *									
≥9 h	−0.01	0.02	−0.05, 0.03	−0.01	0.03	−0.08, 0.06	−0.02	0.02	−0.05, 0.02
Social jetlag									
<1 h *									
1 to <2 h	−0.01	0.01	−0.04, 0.02	−0.02	0.02	−0.05, 0.01	**−0.11**	**0.03**	**−0.17, −0.06**
≥2 h	−0.03	0.02	−0.06, 0.002	−0.03	0.02	−0.63, 0.003	−0.02	0.02	−0.07, 0.03
Shift work × sleep duration									
Shift work × <6 h				−0.05	0.05	−0.15, 0.04			
Shift work × 6 to <9 h *									
Shift work × 9 h				−0.003	0.04	−0.08, 0.08			
Shift work × social jetlag									
Shift work × <1 h *									
Shift work × 1 to <2 h							**0.15**	0.03	**0.09, 0.22**
Shift work × ≥2 h							−0.01	0.03	−0.08, 0.05

* = reference group; Note: adjusting for age, sex, race, education, marital status, ratio of family income to poverty, physical activity, BMI, smoking, and self-reported healthy diet; * Numbers in bold indicate significant at *p* < 0.05.

## Data Availability

The data used in this study are derived from the National Health and Nutrition Examination Survey (NHANES), which is publicly available and can be accessed through the Centers for Disease Control and Prevention (CDC) website. NHANES datasets are freely accessible to researchers and the public at the following link: NHANES Data Access.
